# P7170, a novel inhibitor of mTORC1/mTORC2 and Activin receptor-like Kinase 1 (ALK1) inhibits the growth of non small cell lung cancer

**DOI:** 10.1186/1476-4598-13-259

**Published:** 2014-12-02

**Authors:** Venkatasubbaiah A Venkatesha, Asavari Joshi, Magesh Venkataraman, Vinay Sonawane, Dimple Bhatia, Prashant Tannu, Julie Bose, Sarika Choudhari, Ankita Srivastava, Prashant Kumar Pandey, Vaibhavi J Lad, Ramachandra Sangana, Tausif Ahmed, Anagha Damre, Vijaykumar Deore, Bichismita Sahu, Sanjay Kumar, Somesh Sharma, Veena R Agarwal

**Affiliations:** Piramal Life Sciences Ltd, # 1 Nirlon Complex, Off: Western Express Highway, Goregaon (East), Mumbai, Maharashtra 400063 India

**Keywords:** NSCLC, mTORC1, mTORC2, STAT3, PI3K, EGFR-TKI, Tumor xenograft, Targeted therapeutics, PK-PD

## Abstract

**Background:**

Lung cancer is the major cause of cancer-related deaths and many cases of Non Small Cell Lung Cancer (NSCLC), a common type of lung cancer, have frequent genetic/oncogenic activation of *EGFR*, *KRAS*, *PIK3CA*, *BRAF*, and others that drive tumor growth. Some patients though initially respond, but later develop resistance to erlotinib/gefitinib with no option except for cytotoxic therapy. Therefore, development of novel targeted therapeutics is imperative to provide improved survival benefit for NSCLC patients. The mTOR cell survival pathway is activated in naïve, or in response to targeted therapies in NSCLC.

**Methods:**

We have discovered P7170, a small molecule inhibitor of mTORC1/mTORC2/ALK1 and investigated its antitumor efficacy using various *in vitro* and *in vivo* models of human NSCLC.

**Results:**

P7170 inhibited the phosphorylation of AKT, S6 and 4EBP1 (substrates for mTORC2 and mTORC1) levels by 80-100% and growth of NSCLC cells. P7170 inhibited anchorage-independent colony formation of NSCLC patient tumor–derived cells subsistent of disease sub-types. The compound also induced apoptosis in NSCLC cell lines. P7170 at a well-tolerated daily dose of 20 mg/kg significantly inhibited the growth of NSCLC xenografts independent of different mutations (*EGFR*, *KRAS*, or *PIK3CA*) or sensitivity to erlotinib. Pharmacokinetic-pharmacodynamic (PK-PD) analysis showed sub-micro molar tumor concentrations along with mTORC1/C2 inhibition.

**Conclusions:**

Our results provide evidence of antitumor activity of P7170 in the erlotinib –sensitive and –insensitive models of NSCLC.

**Electronic supplementary material:**

The online version of this article (doi:10.1186/1476-4598-13-259) contains supplementary material, which is available to authorized users.

## Background

Lung cancer remains the leading cause of cancer related deaths worldwide. Eighty-five percent of the lung cancer cases are presented as Non-Small Cell Lung Cancers (NSCLC) in contrast to fifteen percent of Small Cell Lung Cancers (SCLC), and typically seventy-five percent of NSCLC new cases are being diagnosed at late advanced disease stage (ACS, Surveillance Research, 2013; [1]). Current research suggests that the importance of several molecular signaling pathways in Non-Small Cell Lung Cancer (NSCLC) cells that promote tumor growth. These include, but are not limited to activating mutations or amplification of *EGFR*, *KRAS*, *PIK3CA*, *BRAF*, and *EML4-ALK* gene rearrangements and loss of *PTEN*[2–4].

The frequencies of activating mutations of *EGFR* and/or *KRAS* in NSCLC varied in different studies (8–60%) depending on the patient selection biases [5–7]. Recently, in a large and unselected cohort prospective screening of newly diagnosed 552 NSCLC patients, the *EGFR* mutation rate was found to be only 4.9% [8]. Despite an improved PFS (progression free survival) with EGFR-TKI (tyrosine kinase inhibitor) that effectively targets mutant *EGFR* avidly than wild type, the overall survival remained controversial [9, 10]. These findings suggest a possible role of other molecular pathways in the NSCLC disease progression. A retrospective study of patients showed that *KRAS* mutation with or without *EGFR* copy number alteration could predict chances of NSCLC disease progression [11]. Blocking RAS-RAF-MEK-ERK cell growth pathway that channelizes signals from upstream EGFR, KRAS, and BRAF [12–14] has been shown to be important in treating NSCLC. In addition, constitutive activation of AKT has emerged as a mechanism of cell survival and/or resistance to chemotherapy and radiation in NSCLC [15]. Utilization of ErbB-3 signaling in response to gefitinib in gefitinib-sensitive cells and IGFIR signaling in gefitinib-resistant cells was shown as a compensatory mechanisms that result in the activation of phosphoinositide 3-kinase (PI3K) in EGFR wild type NSCLC cells [16, 17]. Also, cooperative up regulation of PI3K and mammalian Target Of Rapamycin (mTOR) pathways in NSCLC patient specimens with or no *EGFR* mutations suggested the importance of PI3K-mTOR signaling in NSCLC [18–20]. Additionally, suppression of PI3K-mTOR pathway has shown to be effective in inhibiting the growth of KRAS mutant NSCLC tumors in a mouse model [21]. Hyper activation of mTOR signaling frequently occurs in nearly 70% of patient tumors and because mTOR regulate eukaryotic cellular functions such as cell growth, cell survival, metabolism, response to stress, translation, and transcription through multiple pathways [22], several mTOR inhibitors are being discovered and evaluated for cancer therapy. It is now understood that both mTORC1 and mTORC2 activity is essential for growth of a subset of tumors by activating 4EBP1/ribosomal S6 and AKT respectively, hence an inhibitor for the same remain needed. Therefore, we developed an mTOR pathway inhibitor P7170 that showed potent inhibitory activity on mTORC1, mTORC2, and ALK1. [A separate manuscript under revision in the journal, Molecular Cancer Therapeutics; AACR 2012 conference posters: Agarwal VR et al., Can Res, 2012, 72(8 Supplement): Abstract no 3742 and 3759]. In this report we provide evidence for its efficacy in patient tumor-derived lung cancer cells *in vitro* and in mouse models of erlotinib-sensitive and -insensitive NSCLC cell line-derived xenografts. The chemical structure of P7170 is included in the patent # WO-2012007926A1.

## Results

### P7170 inhibited mTOR signaling

**Figure 1:**
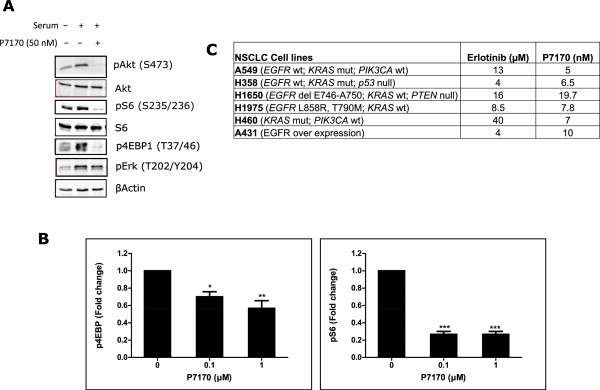
**P7170 inhibited PI3K/mTOR signaling and the growth of erlotinib-resistant NSCLC cell lines. (A)** Exponentially growing H460 cells were seeded in petridishes (2 × 10^6^ cells/dish) and after cell attachment overnight, cells were serum starved for 14-16 h. Cells were then treated for 1 h with P7170 followed by 0.5 h incubation in the presence of 20% fetal bovine serum. Cells were trypsinized and lysed before electrophoresis of cellular extracts for western blot analyses of proteins of interest. **(B)** Quantification of pS6 and p4EBP1 protein levels in H460 cells after acquisition and analyses of immunofluorescence signals in the Cellomics Array scan platform. H460 cells were seeded in 96-well plates and treated with 0.1 or 1 μM of P7170 for 16 h; drug-containing media was removed and the cells were fixed and stained with antibodies to phosphorylated form of human S6 and 4EBP1 proteins and secondary antibodies conjugated to DyLight 549. Statistical significance values are as follows: *p < 0.05, **p < 0.01, ***p < 0.001. **(C)** IC_50_ of erlotinib or P7170 in various NSCLC cell lines after 48 h of treatment.

We evaluated the activity of P7170 in cell based assays. The phosphorylation of AKT (S473) (substrate of mTORC2) [23], S6 (indirect substrate of mTORC1), and 4EBP1 (substrate of mTORC1) were nearly completely inhibited (100%) in H460 NSCLC cells upon treatment with 50 nM P7170. In the same experiment, phosphorylation of ERK, the effector of RAF-MEK-ERK pathway was marginally decreased (10%) (Figure 1A). The kinase activities of upstream PI3K alpha and mTOR were inhibited by P7170 (IC_50_ = 2.2 and 4.4 nM, respectively) but potent biochemical activity of PI3K did not translate in intact cells most likely because of feedback mechanism of mTOR inhibition. P7170 is a weak PI3K inhibitor (a separate manuscript submitted). In an immunofluorescence assay, P7170 treatment caused a consistent and marked decrease in the phosphorylation of S6 with a concentration-dependent suppression of p4EBP1 in H460 cells (Figure 1B, Additional file 1: Figure S1). Longer incubation time with P7170 resulted in an enhanced inhibition of pS6 and p4EBP1 (Additional file 1: Figure S1). The effect of P7170 on cell growth was evaluated in three different NSCLC cell lines, where a dose-dependent inhibition was observed. The IC_50_ of P7170 in EGFR over expressing A431 (EGFR wild type) cells was 10 nM compared to 5 and 7 nM in *KRAS* mutant A549 and H460 cell lines, respectively. In general, P7170 showed nano molar IC_50_ concentrations in the growth of various NSCLC cell lines as opposed to micro molar IC_50_ of erlotinib (Figure 1C).

### P7170 inhibited the clonogenic potential of patient tumor derived cancer cells

Tumor specimens used in this study were histologically derived from three sub-types e.g., adenocarcinoma (FA), squamous cell carcinoma (FE), or large cell carcinoma (FL) where, the tumor cells derived from human xenografts were designated with a prefix ‘LX’ (Table 1). Although the mutation status of *EGFR* or other key genes in these patient tumor-derived cells is unknown, the status of *KRAS*, *PIK3CA*, or *PTEN* was mostly wild type (Table 1). To examine the effect of P7170 on the clonogenic activity, tumor cells obtained from 13 different lung cancer patient biopsies were treated with P7170 (0.01 nM–1 μM) followed by monitoring their growth in soft-agar. The concentration that resulted in the 50% attenuation of colony formation (IC_50_) of P7170 was computed as described in Table 1 and Additional file 2: Figure S2. The IC_50_ values for P7170 were in the broad range of 3 to 300 nM independent of either the genetic status of *KRAS*, *PIK3CA*, *PTEN* or the tumor sub-types (Table 1).

**Table 1:** **P7170 inhibits the growth of NSCLC patient-derived cancer cells**

Tumor designation	Model #	Histology	Kras	PIK3CA	PTEN	IC50 (nM)
LXFA	1012	adenocarcinoma	wt	wt	wt	45
LXFA	1584	adenocarcinoma	NA	NA	NA	2
LXFA	586	adenocarcinoma	wt	wt	wt	9
LXFA	629	adenocarcinoma	wt	wt	wt	5.5
LXFA	677	adenocarcinoma	wt	wt	wt	300
LXFA	737	adenocarcinoma	wt	wt	wt	300
LXFA	749	adenocarcinoma	wt	wt	wt	17
LXFE	1422	Squamous cell carcinoma	wt	wt	wt	8.5
LXFE	470	Squamous cell carcinoma	wt	E545K heterozygous	wt	50
LXFE	646	Squamous cell carcinoma	wt	wt	wt	100
LXFL	1674	Large cell	G12C homozygous	wt	wt	30
LXFL	430	Large cell	wt	wt	wt	3
LXFL	529	Large cell	wt	wt	wt	50

Various patient tumor xenograft-derived NSCLC cells treated with P7170 at log concentrations (range: 0.01 nM – 1 μM) followed by growth in soft-agar to evaluate colony formation. Description of patient’s tumor histology sub-type, mutation status, and IC_50_ of P7170 in these tumor xenograft-derived cells by soft-agar colony formation is shown.

### P7170 caused apoptosis and STAT3 inhibition in cancer cell lines

**Figure 2:**
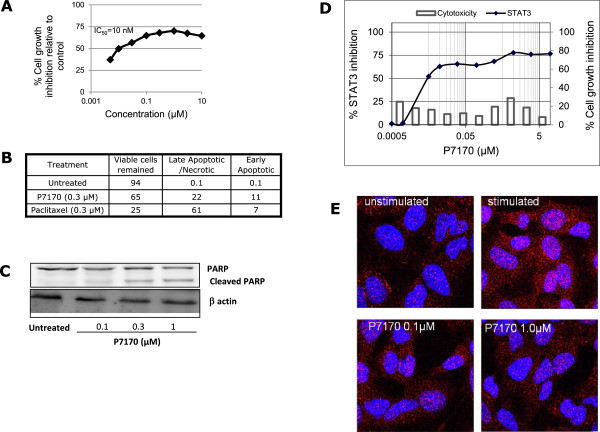
**P7170 induced apoptosis and decreased STAT3 activity in NSCLC cells. (A)** Cell growth inhibition of exponentially growing H460 NSCLC cells by P7170. **(B)** Annexin V and propidium iodide positive cells analyzed by flow cytometry after 16 h of treatment. **(C)** H460 cells were seeded in 90 mm Petri dishes (1 × 10^6^ cells/ plate). The cells were serum starved for 16 h. Fresh medium was added to the plates and treated with P7170 (0.1, 0.3, and1 μM) for 24 h; total cellular proteins were processed for western blotting for cleaved PARP; **(D)** Inhibition of STAT3 activity performed using Panomics STAT3 luciferase kit, corresponding cell toxicity at same concentrations of P7170 was determined by PI-based assay (plotted on the secondary axis on right side of the panel). **(E)** Immunofluorescence staining for STAT3 phosphorylation in A549 NSCLC cells: pSTAT3 (red) and nucleus (blue). See Methods for details.

To further understand the mechanism of cell growth inhibition, H460 cells were treated with P7170 for 24 h and analyzed by flow cytometry for propidium positive dead cells. A significant increase in dead cells upon treatment with P7170 was observed at 10 nM compared to untreated control (p < 0.05; Figure 2A). In the Annexin V-propidium iodide apoptosis assay, we found that treatment with 300 nM P7170 resulted in a 35% loss of cell viability with 22% cells in late apoptosis/necrosis, and 11% cells in early apoptosis indicating the onset of cell death (Figure 2B; Additional file 3: Figure S3). The onset of apoptosis correlated with cell growth inhibition at similar concentrations (100 – 300 nM) and at the same incubation time of 24 h (Figure 2A). To ascertain whether apoptotic response by P7170 involves DNA damage or elicited DNA damage-repair response, PARP cleavage, a widely used marker for measuring DNA-damage repair response was examined. In a separate experiment, treatment of H460 cells with an increasing concentration of P7170 was found to induce PARP cleavage (Figure 2C).

Since P7170 also inhibited JAK2 kinase activity (IC_50_: 39 nM, manuscript submitted), we evaluated STAT3 activation, a target downstream of JAK2 because similar to PI3K pathway, STAT-family members are activated upon phosphorylation (at different Serine/Tyrosine residues) by cytokines and growth factors. Using a cell based STAT3-reporter assay, P7170 demonstrated a marked inhibition of transcriptional activation of STAT3 in HeLa cells (p < 0.01) with minimal (18%) cytotoxicity (IC_50_: 4.7nM, Figure 2D). Also, in an immunofluorescence assay in A549 NSCLC cells, there was a prominent decrease in the nuclear staining for phosphorylated STAT3 (Y705) after treatment with 0.1–1 μM P7170 (Figure 2E).

### *In vivo*anti-tumor efficacy of P7170 in mice bearing NSCLC xenografts

**Figure 3:**
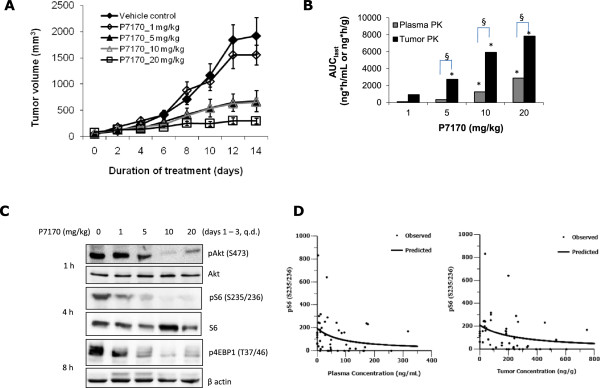
**Anti-tumor efficacy of P7170 correlated with tumor pharmacokinetic and pharmacodynamic parameters. (A)** Tumor volume (mm^3^) of H460 xenografts in SCID mice was measured in P7170 treatment groups twice weekly. **(B)** For pharmacodynamic studies, P7170 was administered into tumor bearing mice once daily consecutively for 3 days. Plasma and tumors samples were collected at 1, 4, 8, 24 h post-P7170-last dose (minimum 3 mice per time point) for estimating P7170 concentrations by LC-MS/MS. P7170 concentrations for each dose (1, 5, 10, or 20 mg/kg) were calculated based on the AUC (area under curve) constructed using concentrations at 1, 4, 8, 24 h post-P7170-last dose. Statistical significance: *p < 0.05 **(C)** Representative protein western blots showing response to the treatment in the experiment **(B)**. **(D)** In the same experiment as in **(B)**, part of tumor tissues was processed for western blotting analyses of proteins using specific antibodies; protein band intensities were normalized to respective loading controls. The band intensities of pS6 were plotted against the absolute plasma or tumor P7170 concentrations. The correlations of pS6 (S235/236) levels to plasma or tumor P7170 concentrations were performed using the non-compartmental analysis tool of WinNonlin Professional version 6.1: Predicted curve represents the trend in protein level derived from each tumors sample (closed circles) with respect to tumor concentrations of P7170.

In order to understand the antitumor efficacy of P7170 in erlotinib-sensitive and insensitive *in vivo* models, subcutaneous xenografts of NSCLC, using H1975, H1650, or H460 cell lines were established. Although H1650 and H1975 cell lines are equally responsive to erlotinib *in vitro*, literature [24, 25] suggests that H1975 xenografts are erlotinib-resistant. Treatment with P7170 resulted in a significant growth inhibition (70% TGI, p < 0.001, Table 2) of xenograft derived from H1650 erlotinib-sensitive cells [*EGFR* del E748-A750 (activating mutation); *KRAS* mutant; *PIK3CA* wild type] at 5 mg/kg, a dose much lower than its MTD. However, the same dose of P7170 was not very effective in mice bearing erlotinib-insensitive H1975 [*EGFR* T790M, L858R; *KRAS* wild type; *PIK3CA* wild type] xenografts, but treatment with a15 mg/kg dose resulted in a significant tumor growth inhibition (92% TGI, p < 0.001, Table 2). Interestingly, P7170 was efficacious (88% TGI at 20 mg/kg; 66% TGI at 10 mg/kg with p < 0.001, Table 2 and Figure 3A) in another model of erlotinib -insensitive H460 (*EGFR* wild type; *KRAS* mutant; *PIK3CA* mutant) xenografted animals. P7170 administration was well tolerated at doses up to 20 mg in different xenograft models (Additional file 4: Figure S4). These results provide evidence for the efficacy of P7170 in erlotinib-sensitive, and –insensitive NSCLC irrespective of known genetic mutations in these cells.

**Table 2:** **P7170 decreased growth of NSCLC cell line-derived tumor xenografts**

Percent tumor growth inhibition in NSCLC-derived xenografts			
P7170 ( ***p.o., QD*** )	H1650	H1975	H460
5 mg/kg	70% (p < 0.001)	45% (p < 0.01)	53% (p < 0.001)
15 mg/kg		92% (p < 0.001)	66% (p < 0.001)
20 mg/kg			88% (p < 0.0001)
Duration of treatment	15 days	13 days	11 days
Tumor volume at the beginning of treatment ~ 100 mm3			

Nude mice bearing xenografts of human NSCLC cell lines H1650, H1975, or H460 were treated with P7170 at indicated doses for 10 – 15 days (*p.o., QD*). All mice maintained body weight with a loss not more than 10%.

### *Pharmacokinetic-pharmacodynamic correlation of P7170*in human H460 NSCLC xenograft model

H460 xenograft model was chosen for this study. Tumor bearing mice were treated with different doses of P7170. Administration of P7170 at a 20 mg/kg dose resulted in 88% tumor growth inhibition (Figure 3A) confirming the results of a previous experiment (Table 2). Tumor growth inhibition with 5 and 10 mg/kg doses was significant compared to the vehicle (~53 and 66% respectively, p < 0.05) but did not differ much between the doses. In the efficacy study (Figure 3A); plasma and tumor samples collected at 1, 4, 8, or 24 h post P7170-last dose were analyzed to determine the concentrations of P7170 by LC-MS. The results of this experiment indicate a dose dependent increase in P7170 concentrations in the tumors based on area under the curve (AUC) (*p < 0.05 between two doses for plasma or tumor P7170 concentrations independently; ^§^p < 0.05 is the significance when concentrations of P7170 in the plasma and tumor were compared; Figure 3B). Also, P7170 concentrations were 2.7-fold higher in tumor versus plasma in 20 mg/kg group.

In a separate PK-PD study, H460 xenograft-bearing mice were treated with P7170 (1, 5, 10, or 20 mg/kg/day) consecutively for 3 days. Plasma and tumor samples were collected at 1, 4, 8, and 24 h after the administration of the last dose for PK-PD-analyses. Based on the time to achieve Cmax, plasma and tumor concentrations of P7170 were pooled from two time points (1 and 4 h) in each group (Figure 3B). Tumor to plasma concentrations revealed a preferential accumulation of P7170 in the tumor than plasma in a dose-dependent manner (3 – 11-fold) (Figure 3B). To ascertain the pharmacodynamic effect of P7170 in tumor xenografts, western blotting of key proteins in the PI3K-Akt-mTOR pathway in tissue extracts was performed, and the representative protein blots derived from the samples collected during 1-8 h post P7170-last-dose are shown (Figure 3C). AKT phosphorylation (S473) the direct substrate of mTORC2 was found to be strongly inhibited in response to 10 or 20 mg/kg treatment. Also, the phosphorylation of ribosomal S6 (S235/236) and 4EBP1 (T37/46), targets downstream of mTOR were inhibited by 5-20 mg/kg doses (Figure 3C). For the PK-PD-analyses, the normalized individual protein band intensities (pAKT, pS6, or p4EBP1) were plotted against P7170 concentrations in respective tumor samples. PK-PD analyses were carried out based on Inhibitory E_max_ models (Additional file 5: Table S1) using Phoenix WinNonlin software (version 6.1) to evaluate the correlation between the level of tumor biomarkers (pAKT, pS6, and p4EBP1) and plasma and tumor concentrations of P7170. A trend showing a decrease in pS6 levels with increasing tumor and plasma P7170 on day 3, indicates target pathway engagement (Figure 3D). No correlations were observed for pAKT and p4EBP1 (Additional file 6: Figure S5). Additional details for PD model and parameter estimates are presented in Additional file 5: Table S1.

## Statistics

Results are presented as mean ± standard deviation. Statistical significance was determined using the two-way ANOVA with Turkey’s post hoc test. Data with p < 0.05 were considered significant. Statistical analysis was done using GraphPad Prism, version 5.0c.

## Discussion

In cancer, majority of growth factor receptor protein tyrosine kinases such as EGFR, HER2/3, FGFR, PDGFR, IGFR were found to be hyper activated. The important downstream pathways that are instrumental in regulating cell survival include, PI3K/mTOR and RAS-ERK. Many PI3K-AKT inhibitors have not produced the expected responses in patients with advanced cancers at tolerated doses. Benefit from AKT inhibition is limited due to mTORC2-mediated reactivation of PI3K-AKT signaling [23]. As mTOR is frequently hyper activated in human tumors and because the activity of both mTORC1 and mTORC2 complexes is essential for growth of a subset of tumors we aimed to develop an inhibitor for both these complexes.

We describe here the antitumor efficacy of an agent P7170 attributed to inhibition of mTORC1/mTORC2/ALK1, and JAK-STAT3 signaling in NSCLC. We show that treatment with P7170 results in a marked reduction in the survival of NSCLC cells with different mutations in *EGFR*, *KRAS*, *PIK3CA*, or *PTEN* and inhibition of tumor growth *in vivo*. Supported by its pharmacodynamic effects (inhibition of phosphorylation of mTOR pathway proteins such as AKT, ribosomal S6, and 4EBP1) development of P7170 provides an attractive opportunity as a treatment option for NSCLC.

Consistent with reports suggesting a critical importance of mTOR pathway for the clonogenic cell growth [26], we found P7170 to inhibit colony formation of various patient-derived NSCLC cell lines in the soft-agar assay. Inhibition of mTOR signaling contributed to increased cellular apoptosis (PARP cleavage and Annexin V positive cells) with concomitant decrease in the growth of H460 cells. The decreased cell growth by P7170 could have resulted in inability of cells to colonize. An IC_50_ concentration of P7170 (10 nM) where 50% of cells did not undergo apoptosis indicated other mechanisms may be involved mediating cell death. To delineate this, we found studies reporting a persistent activation of STAT3 (pSTAT3) in nearly 50% of lung adenocarcinomas [27]. It is known that JAK2 directly regulate STAT3 activation [28], and since P7170 is known to have activity against the JAK2 kinase, it was hypothesized that P7170 may inhibit STAT3 activation. As expected, P7170 inhibited STAT3 activity in a luciferase expression system in HeLa cells. In an immunofluorescence assay, P7170 has also inhibited STAT3 phosphorylation (Y705) *in situ* in A549 NSCLC cells. In understanding the functional effect of these results, a study showed that inhibition of STAT3 was responsible for NSCLC tumor growth suppression [29] suggesting that inhibition of JAK2-STAT3 could be a mechanism for cellular apoptosis in P7170 treated cells.

We have demonstrated the *in vivo* antitumor efficacy of P7170 in xenograft models of different erlotinib-sensitive, and –resistant NSCLC cell lines at tolerated doses, and particularly, in xenografts derived from EGFR T790M, L858R erlotinib-resistant cells. The results of the present PK/PD study show mTORC1 and mTORC2 target engagement with a negative correlation between tumor volume and plasma/tumor P7170 concentrations. With the patient treatment in perspective, second generation EGFR TKI or combination with mTOR inhibitor for NSCLC patients with *EGFR* T790M mutation [30] could potentially become options. Although therapeutic options for NSCLC patients with *KRAS* mutation is lacking, new approaches in the clinical testing such as dual MEK and mTOR inhibition or cytotoxic drugs [31] are being evaluated. Therefore, targeting of mTOR cell survival/growth pathways in subjects with erlotinib –sensitive and –insensitive NSCLC might be beneficial. In summary, P7170 showed a target engagement, resulted in tumor cell killing leading to tumor growth inhibition *in vivo*.

## Conclusions

Taken together, our results demonstrate that P7170 has NSCLC cell killing activity via inhibition of mTORC1/mTORC2 and JAK2-STAT3 signaling. This activity resulted in tumor growth inhibition as a single agent in the *in vivo* xenograft models. Therefore, we propose that P7170 could produce benefit in patients with erlotinib-sensitive, and –insensitive NSCLC.

## Methods

### Cell lines and culture

A549, H358, H1975, H1650, and H460 cell lines were purchased from ATCC and maintained in RPMI1640, supplemented with 10% heat inactivated FBS (Gibco). STAT3 Reporter HeLa Stable Cell Line (stably transfected with Luciferase gene under the control of STAT3 promoter) was purchased from Panomics, USA and was maintained in DMEM supplemented with 10% FBS under hygromycin B (Sigma H3274) selection condition. Experimental culture conditions for patient tumor derived cells are given below in methods.

### Cell growth inhibition assay

#### Propidium iodide assay

Exponentially growing cells were plated in 96-well plate 24 h before treatment with P7170. After 48 h of incubation plates were washed with 1× PBS followed by addition of PI-containing PBS (7 μg/ml) and storage at -80°C. After thawing the plate PI fluorescence was measured in POLAR STAR OPTIMA (BMG Lab technologies). Cell Growth Inhibition was normalized to the controls.

#### Soft-agar colony formation of patient tumor-derived xenograft cells

P7170 was evaluated at log concentrations from 0.01 nM to 1 μM in a 14-day soft agar colony formation assay. The Patient tumor explants are passaged as subcutaneous xenograft in NMRI nu/nu mice. For the clonogenic assay, cells were isolated from tumor xenografts (also referred to as patient derived tumor xenografts, PDX) of 13 NSCLC patient specimens established in mice under sterile conditions followed by mechanical disaggregation and subsequent incubation with an enzyme cocktail [consisting of collagenase type IV (41 U /ml), DNase I (125 U /ml), hyaluronidase type III (100 U /ml) and dispase II (1.0 U /ml) in RPMI 1640 medium] at 37°C for 45 minutes. Cells were allowed to pass through sieves of 200 μm and 50 μm sterile nylon mesh and washed twice with sterile PBS buffer. The percentage of viable cells is determined in Neubauer Hemocytometer using trypan blue exclusion. The bottom layer consisted of 0.2 ml/well Iscove’s Modified Dulbecco’s Medium (Invitrogen), supplemented with 20% (v/v) fetal calf serum (Sigma), 0.01% (w/v) gentamicin (Invitrogen) and 0.75% (w/v) agar (BD Biosciences). Tumor cells were added to 0.2 ml of the same culture medium supplemented with 0.4% (w/v) agar and plated in 24-multiwell dishes onto the bottom layer. P7170 at desired concentration in 0.2 ml culture medium was overlaid on the bottom layer. Every dish included six untreated control wells and drug treated groups in triplicate at 6 concentrations. Cultures were incubated at 37°C and 7.5% CO_2_ in the humidified atmosphere for up to 20 days and monitored closely for colony growth using an inverted microscope. Within this period, *in vitro* tumor growth leads to the formation of colonies with diameter > 50 μm. At the time of maximum colony formation, counts were performed with an automated image analysis system (OMNICON 3600, Biosys GmbH). 24 h prior to evaluation, vital colonies are stained with a sterile aqueous solution of 2-(4-iodophenyl)-3-(4-nitrophenyl)-5-phenyltetrazolium chloride (1 mg/ml, 100 μl/well). IC_50_ values were calculated by two point curve fit [(conc. Of inhibitor) versus response (%T/C)] using Oncotest in house database system) or nonlinear regression [log (conc. of inhibitor)] versus response (% T/C) using Graph pad Prism 5 for windows, version 5.01, Graph pad software Inc. CA). For calculation of mean IC_50_ values the geometric mean is used. Results are presented as mean graph plots or heat maps (individual IC_50_ values relative to the geometric mean IC_50_ value) over all cell lines as tested.

### Immunoblotting

Whole cell extracts from treated cell lines or xenografts were prepared using cell lysis buffer [a mixture of protease inhibitor cocktail (Sigma, Cat #P8340) and phosphatase inhibitors (40 mM Beta-glycerol phosphate (Sigma, Cat #G9422), 4 mM DTT (MP Biomedicals, Cat #194821), 0.4 mM NaF (Sigma Cat #S1504), 0.4 mM Sodium-orthovanadate (Sigma, Cat #S6508)]. Total protein concentration from the soluble fraction was determined by Bradford’s method. Equal amount of total protein was resolved on SDS-PAGE gels; protein bands were electro-transferred onto PVDF membrane. Antibodies for immunoblotting are from Cell Signaling Technology [pAKT (S473) (#9271); AKT (#9272); pS6 (S235/236) (#2211); S6 (#2217); p4EBp1 (T37/46) (#9459); pERK (T202/Y204) (#9101); ERK (#4695)]; and from Sigma (Actin #A2228). SuperSignal West Femto (Thermo Scientific Cat #34096) was used for chemiluminescence detection and the signals were captured in the ChemiDoc XRS image system (Bio-Rad). The protein western band intensities were calculated using NIH Image-J software.

### Immunofluorescence

#### pS6 and p4EBP1 staining

H460 cells were seeded in 96-well Black/clear plates (Nunc) followed by treatment on the next day with P7170 for 1 h. Cells were fixed with 3.7% PFA in 1× PBS at RT for 20 min, cell membranes permeabilized with 0.1% Triton X-100 for 90 sec, followed by blocking with 5% BSA (Sigma-Aldrich Cat #A7030) (w/v) in 1 × PBS for 2 h before immunostaining. Primary antibodies used were pS6 and p4EBP1 (Cell Signaling) at dilution (1:500) at RT for 1 h. Secondary Goat Anti-Rabbit antibody IgG (H + L) DyLight 549 Thermo Scientific Cat #35557) probed at 1:1000 dilution for 1 h. Nuclei were stained with Hoechst 33342 (AnaSpec Inc. Cat #83218). Plates were scanned on Cellomics Array Scan VTI HCS Reader.

#### pSTAT3 staining

Exponentially growing A549 cells were seeded on coverslips in a 24 well plate (Nunc) in complete growth media for 14-16 h followed by culture in no serum media for another 16-20 h. Serum starved cells were treated with P7170 (0.1-1 μM) for 1 h followed by stimulation with 30 ng/ml rhIL-6 (R&D systems Cat. No. 206-IL) for 20 min. Cells were washed with 1× PBS, fixed and permeabilized as described earlier. Primary antibodies used were pSTAT3^(Y705)^ (Cell Signaling Cat #9131) incubated (1:100 dilution) at RT for 1 h. Secondary antibody and nuclear staining performed as earlier. Cell images were captured by confocal microscopy (Leica, LSM700) with ZEN2009 software.

### Annexin V – FITC staining and flow cytometry

Exponentially growing H460 cells were seeded in 6 well plate, treated 16 h later with P7170 at 0.3 μM for 24 h. Cells were harvested by trypsinization and processed for FITC Annexin V staining as per manufacture’s protocol (BD Pharmingen Cat #556420). The samples were acquired by FACSCalibur flow cytometer and the signal intensities were analyzed using CellQuest Pro software (Becton Dickinson).

### STAT3 luciferase assay

STAT3 Reporter HeLa cells were seeded (2 × 10^4^ cells/well) in a 96- well white plate (Nunclon Cat #167008). After 16 h, cells were treated for 1 h with P7170 followed by stimulation with 100 ng/ml Oncostatin M (Calbiochem Cat #496260) for 7 h. Cells were lysed in a buffer (a mixture of 125 mM Tris phosphate buffer, 10 mM EDTA, 10 mM DTT, 50% glycerol, 5% Triton ×100) before the addition of luciferin (Promega Cat #E160E), ATP (Sigma Cat #A2383), and coenzyme A (Sigma Cat #C3019). The luminescence generated was measured using POLARstar OPTIMA (BMG Lab technologies). Cytotoxicity due to drug treatment (total 8 h) was determined using propidium iodide.

### *In vivo*xenograft models of NSCLC cell lines and treatment

Nude mice (male, ~6 wks.) from Harlan Labs were housed in animal isolators (Harlan Inc.) with 12 h light dark cycle, 55-75% relative humidity at 22-25°C of room temperature, given access to autoclaved rodent diet (National Centre for Laboratory Animal Sciences, Hyderabad, India) and water *ad libitum*. Animals were acclimatized for a week before implanting subcutaneously with 5×10^6^ NSCLC cells (0.2 ml/site with BD matrigel) in the right flank. When the mean xenograft size reached ~100 mm^3^, mice were randomized into the study groups (n = 9/group). P7170 was suspended in 0.25% CMC +0.1% Tween-80 for administering via oral (*p.o.*) route. Animal body weight and physical signs were monitored throughout the experiment every day. The tumor size was measured with calipers 2-3 times/wk. and the tumor volumes were calculated using the following formula: (length × width)^2^ × 0.5.

### Pharmacokinetic (PK) and Pharmacodynamic (PD) study

#### PK analyses

Plasma samples or tumor homogenates were processed to extract P7170 followed by determining P7170 concentrations using LC-MS/MS.

#### Tumor homogenate preparation

Tumor samples were diluted 4 times of its weight with homogenization solvent methanol: saline (75:25 v/v) and then homogenized to obtain tissue extracts until a uniform homogenate was obtained.

#### Sample preparation

An aliquot (100 μL) of plasma or tumor homogenate was spiked individually with 10 μL of internal standard (P6569, a compound structurally similar to P7170, 1.0 μg/mL). The samples were vortexed for 10 seconds followed by addition of 1 mL ethyl acetate and further vortexed for 5 min. The samples were then centrifuged at 10000 rpm for 5 minutes at 4°C. Supernatants (800 μL) were transferred to relabeled glass tubes and its solvent portion was allowed to evaporate under nitrogen. The dried residues were reconstituted using 200 μL acetonitrile: water (90:10 v/v) by vortex and centrifuged. The supernatants were analyzed for P7170 by a fit-for-purpose LC-MS/MS method. Pharmacokinetic analyses were carried out at each dose level using the non-compartmental analysis tool of Phoenix WinNonlin software (Version 6.1).

#### LC-MS/MS analysis

The chromatographic LC-MS/MS system consisted a Shimadzu LC pump with an API 4000 triple mass spectrometer (Applied Biosystems/MDS Sciex, Foster City, CA) fitted with a TurboIon- Spray interface. P7170 and P6569 (internal standard) were separated on a Thermo BDS Hypersil C18 column of 100 × 4.6 mm I.D. and particle size of 5 μ. The mobile phase composed of two solvents: Solvent A, 5 mM Ammonium formate in 0.1% formic acid and the Solvent B, Acetonitrile in a ratio of 20:80% v/v, respectively. The isocratic HPLC condition was used for analysis. The samples were introduced into the HPLC column with a SIL-20 AC XR autosampler (Shimadzu) and an integrated HPLC pumping system (Shimadzu LC-20 AD XR) with 5 μL injection volume at a flow rate of 0.8 mL/min for a 3 min run time. The analytes were then eluted at RT 1.45 and 1.50 min for P7170 and P6569, respectively followed by MS detection. Electro-spray ionization in positive ion mode (ESI+) was used for ionization and multiple reaction monitor (MRM) mode was chosen for detection. The precursor-product ions pairs were m/z 528.0 → 461.4 (for P7170), and m/z 504.3 → 437.2 (for internal standard, P6569). The optimized acquisition parameters were: Temperature set at 400°C; Curtain gas (CUR), 30 psi (99.99% Nitrogen); Nebulizer Gas (Gas 1), 60 psi (99.99% Nitrogen); Heater Gas (Gas 2), 50 psi (99.99% Nitrogen); Collision-Activated Dissociation (CAD) Gas: 10 v; Ion Spray Voltage (IS), 5500 v.

The unknown plasma and tumor concentrations of P7170 (unbound and plasma protein bound) were calculated from the calibration standards at 0 to 15000 ng/mL by spiking 10 × standards in blank plasma and tumor homogenates collected from untreated nude mice using linear regression according to the following equation:


Where,

y = Peak area ratio of analyte: internal standard

b = Intercept of the corresponding standard curve

a = Slope of the standard curve

x = Concentration of analyte (ng/mL)

Pharmacokinetic analysis was carried out at each dose level using the non-compartmental analysis tool of WinNonlin Professional version 6.1. Pharmacokinetic parameters were determined from mean plasma and tumor concentrations thus obtained at each time point by non-compartmental analysis using Phoenix WinNonlin Professional version 6.1. Concentrations below limit of quantification (LLOQ = 0.5 ng/mL) were considered as zero for PK analysis. Nominal time points were used for PK analysis.

#### PK-PD analyses

PD analysis was performed to derive a correlation between tumor marker levels (pS6, pAKT and p4EBP1) and the corresponding plasma and tumor P7170 concentrations after dosing the tumor-bearing mice with P7170 once daily for 3 days. Plasma and tumors samples were collected at 1, 4, 8, 24 h post-P7170-last dose for estimating P7170 concentrations by LC-MS/MS. P7170 tumor concentrations for each dose (1, 5, 10, or 20 mg/kg) were calculated based on the AUC (area under curve) constructed using concentrations at 1, 4, 8, 24 h post-P7170-last dose. The normalized phosphoprotein western band intensities of AKT (S473), S6 (S235/236), and 4EBP1 (T37/46) as determined in tumor xenografts (in 1, 4, 8, 24 h post P7170-last dose) of different experimental arms were plotted against corresponding tumor xenograft P7170 concentration using the non-compartmental analysis tool using Phoenix WinNonlin Professional version 6.1. The analyses were performed using various inhibitory E_max_ models available in Phoenix WinNonlin software (version 6.1). The model with the lowest AIC (Akaike Information Criteria) value is selected as the best fit model. If two models had similar AIC values, then selection was made qualitatively by visual inspection of the model fit and % CV of the parameter estimates.

## Electronic supplementary material

Additional file 1: Figure S1: P7170 inhibited PI3K-mTOR signaling. pS6 (S235/236) and p4EBP1 (T37/46) protein levels were determined by immunofluorescence staining in H460 cells. H460 cells were seeded in 96-well plates (black and transparent bottom) before treatment with 0.1 or 1 μM of P7170 for 1 h; drug containing media was removed and the cells were fixed and stained with antibodies to phosphorylated form of human S6 and 4EBP1 proteins and secondary antibodies conjugated to DyLight 549, and signals acquired and analyzed in Cellomics high content array scan reader. (PPTX 2 MB)

Additional file 2: Figure S2: P7170 inhibits the colony formation of tumor cells isolated from Non Small Cell Lung Cancer patients. Dose response curves for various patient tumor xenograft-derived NSCLC cells treated with P7170 in a soft-agar colony formation assay. (PPTX 128 KB)

Additional file 3: Figure S3: Cellular apoptotic analysis after P7170 treatment. In the flow cytometry analysis gating was set using untreated cells (A); Increased cellular apoptosis and necrosis after P7170 treatment (B) or Paclitaxel treatment (C). (PPTX 188 KB)

Additional file 4: Figure S4: Body weight changes of nude mice bearing NSCLC cell line-derived xenografts treated with P7170. (A) Percent body weight changes in the H1650 NSCLC cell-derived xenograft treated with P7170 (see Table 2); (B) Percent body weight changes in the H1975 NSCLC cell-derived xenograft treated with P7170 (see Table 2); Percent body weight changes in the H460 NSCLC cell-derived xenograft treated with P7170 (see Figure 3A). (PPTX 404 KB)

Additional file 5: Table S1: Summary of PK/PD study. Correlation analysis of tumor volume to P7170 concentration. E0 represents the level of biomarker in plasma and tumor at baseline i.e. when the concentration of drug in plasma and tumor is 0 (zero). IC_50_ represents the concentration of drug in plasma and tumor required to produce 50% of the maximal inhibition. (PPTX 64 KB)

Additional file 6: Figure S5: Pharmacodynamic correlation of pAKT (S473) and p4EBP1 (T37/46) with tumor P7170 concentrations. Based on the study described in Figure 3, pharmacodynamic correlations of tumor pAKT (S473) and p4EBP1 (T37/46) levels to the plasma and tumor concentrations of P7170 were performed. The correlation plots calculated using the model (Additional file 5: Table S1): Correlation of tumor pAKT levels with P7170 concentrations in plasma (A) and tumor (B); and correlation of tumor p4EBP1 levels with P7170 concentrations in plasma (C) and tumor (D). (PPTX 146 KB)

## Abbreviations

NSCLC: Non small cell lung cancer
mTOR: Mammalian target of Rapamycin
mTORC1: Mammalian target of Rapamycin complex 1
mTORC2: Mammalian target of Rapamycin Complex 2
EGFR: Epidermal growth factor receptor
TKI: Tyrosine kinase inhibitor
PI3K: Phosphoinositide 3-kinase
KRAS: Kirsten rat sarcoma virus
PK: Pharmacokinetics
PD: Pharmacodynamics
JAK2: Janus kinase 2
STAT3: Signal transducer and activator of transcription 3
TGI: Tumor Growth Inhibition.
