# Supplementary figures and images for: P7170, a novel inhibitor of mTORC1/mTORC2 and Activin receptor-like Kinase 1 (ALK1) inhibits the growth of non small cell lung cancer
Source: Mol Cancer. 2014 Dec 2;13:259. doi: 10.1186/1476-4598-13-259 (PMC4289333; doi:10.1186/1476-4598-13-259)

## Slide 1
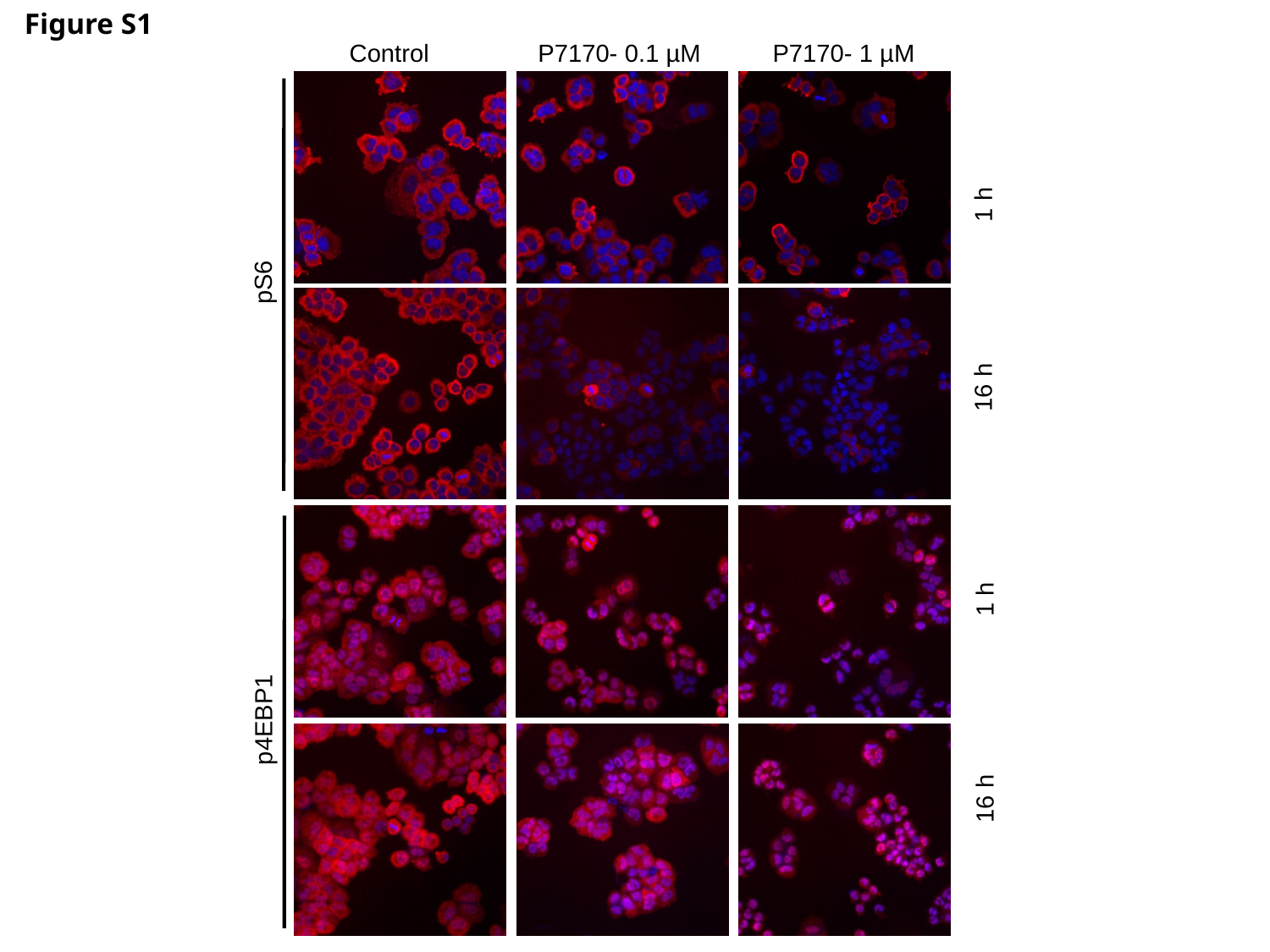

Figure S1
Control
P7170- 0.1 µM
P7170- 1 µM
pS6
1 h
16 h
p4EBP1
1 h
16 h

Supplement: Supplementary file 1 — Additional file 1: Figure S1: P7170 inhibited PI3K-mTOR signaling. pS6 (S235/236) and p4EBP1 (T37/46) protein levels were determined by immunofluorescence staining in H460 cells. H460 cells were seeded in 96-well plates (black and transparent bottom) before treatment with 0.1 or 1 μM of P7170 for 1 h; drug containing media was removed and the cells were fixed and stained with antibodies to phosphorylated form of human S6 and 4EBP1 proteins and secondary antibodies conjugated to DyLight 549, and signals acquired and analyzed in Cellomics high content array scan reader. (PPTX 2 MB) [file 12943_2014_1461_MOESM1_ESM.pptx]

## Slide 1
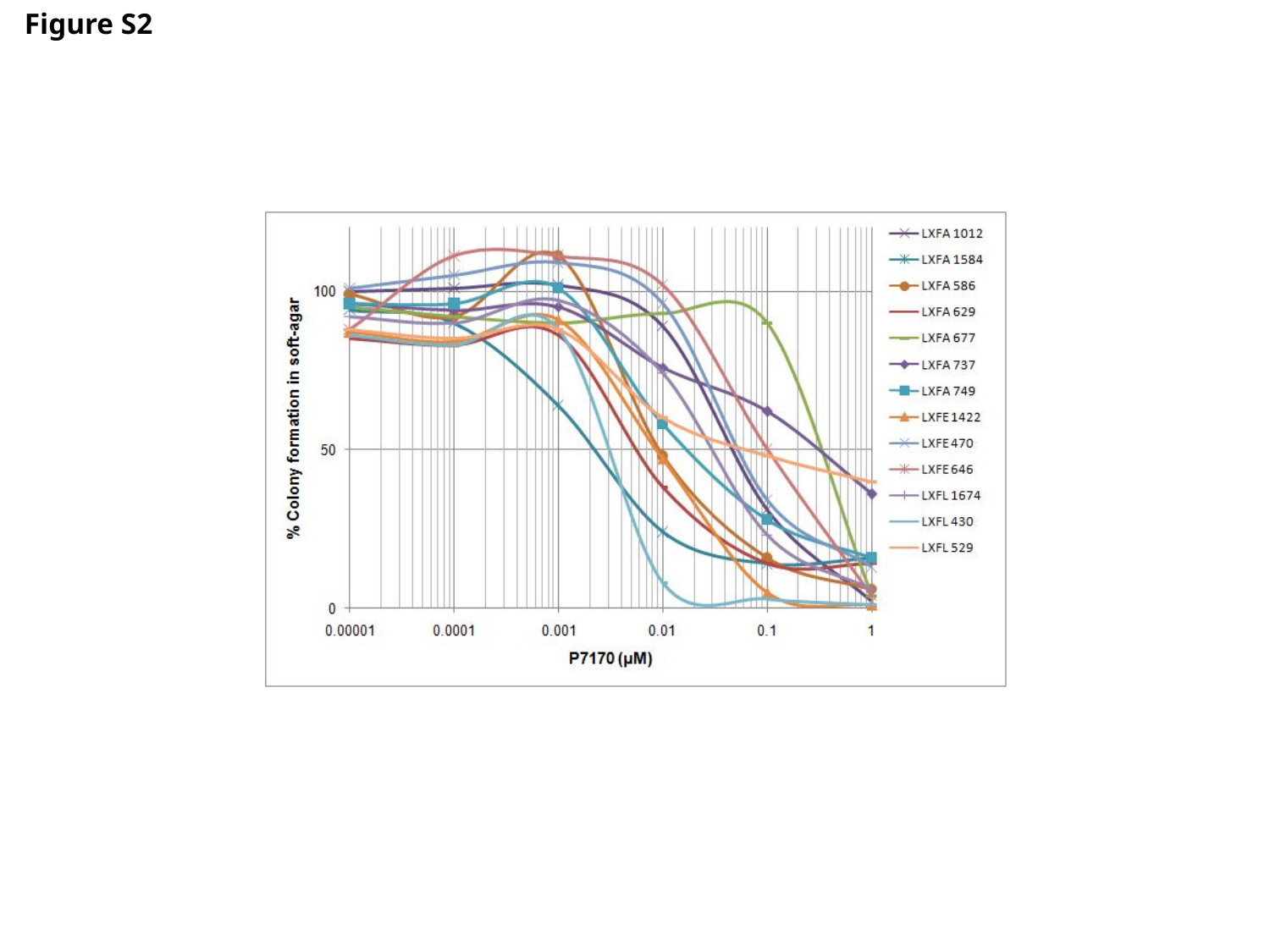

Figure S2

Supplement: Supplementary file 2 — Additional file 2: Figure S2: P7170 inhibits the colony formation of tumor cells isolated from Non Small Cell Lung Cancer patients. Dose response curves for various patient tumor xenograft-derived NSCLC cells treated with P7170 in a soft-agar colony formation assay. (PPTX 128 KB) [file 12943_2014_1461_MOESM2_ESM.pptx]

## Slide 1
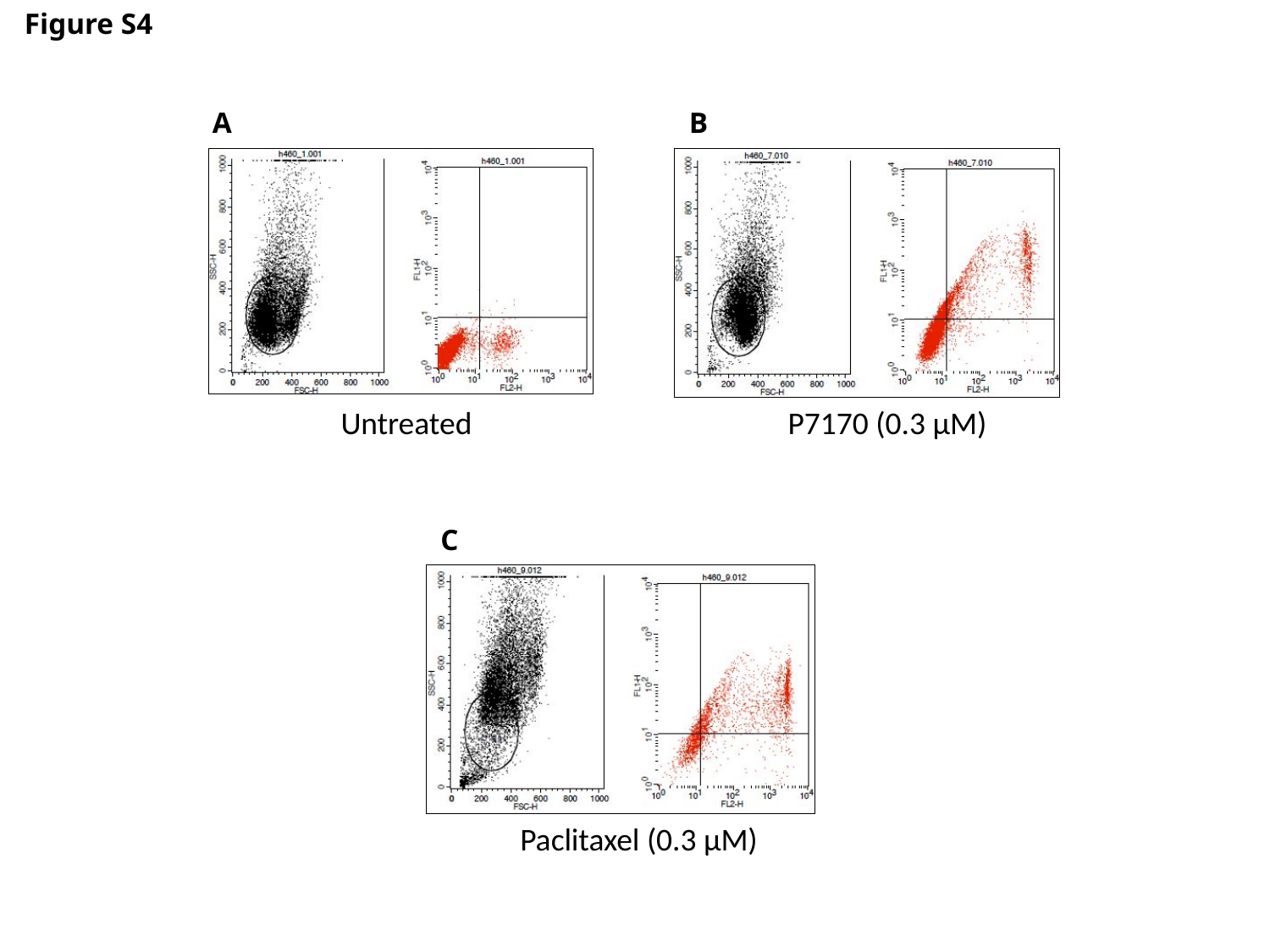

Figure S4
A
B
Untreated
P7170 (0.3 µM)
C
Paclitaxel (0.3 µM)

Supplement: Supplementary file 3 — Additional file 3: Figure S3: Cellular apoptotic analysis after P7170 treatment. In the flow cytometry analysis gating was set using untreated cells (A); Increased cellular apoptosis and necrosis after P7170 treatment (B) or Paclitaxel treatment (C). (PPTX 188 KB) [file 12943_2014_1461_MOESM3_ESM.pptx]

## Slide 1
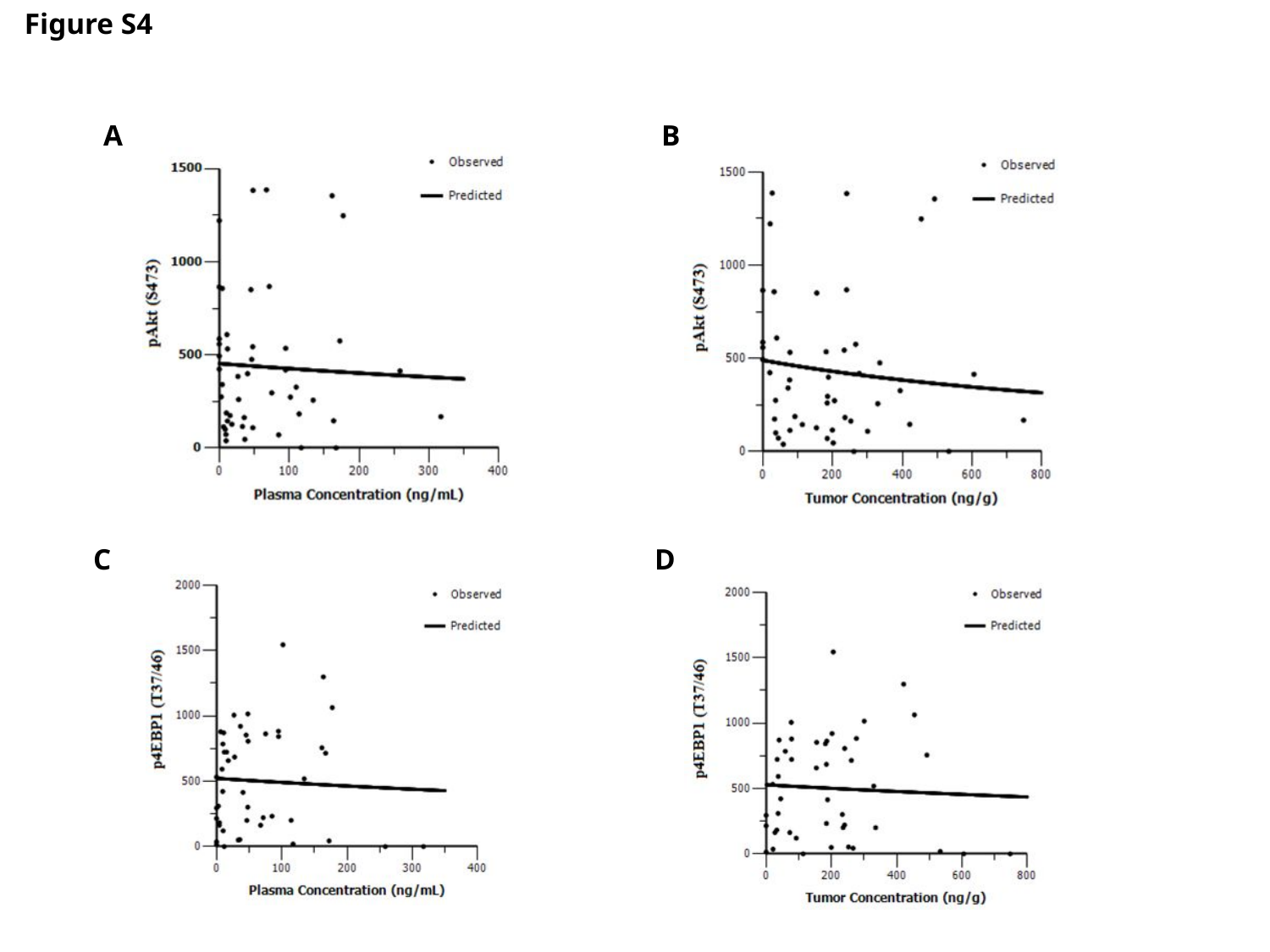

Figure S4
A
B
C
D

Supplement: Supplementary file 6 — Additional file 6: Figure S5: Pharmacodynamic correlation of pAKT (S473) and p4EBP1 (T37/46) with tumor P7170 concentrations. Based on the study described in Figure 3, pharmacodynamic correlations of tumor pAKT (S473) and p4EBP1 (T37/46) levels to the plasma and tumor concentrations of P7170 were performed. The correlation plots calculated using the model (Additional file 5: Table S1): Correlation of tumor pAKT levels with P7170 concentrations in plasma (A) and tumor (B); and correlation of tumor p4EBP1 levels with P7170 concentrations in plasma (C) and tumor (D). (PPTX 146 KB) [file 12943_2014_1461_MOESM6_ESM.pptx]
