# Supplementary material for: P7170, a novel inhibitor of mTORC1/mTORC2 and Activin receptor-like Kinase 1 (ALK1) inhibits the growth of non small cell lung cancer
Source: Mol Cancer. 2014 Dec 2;13:259. doi: 10.1186/1476-4598-13-259 (PMC4289333; doi:10.1186/1476-4598-13-259)
Supplement: Supplementary file 4 — Additional file 4: Figure S4: Body weight changes of nude mice bearing NSCLC cell line-derived xenografts treated with P7170. (A) Percent body weight changes in the H1650 NSCLC cell-derived xenograft treated with P7170 (see Table 2); (B) Percent body weight changes in the H1975 NSCLC cell-derived xenograft treated with P7170 (see Table 2); Percent body weight changes in the H460 NSCLC cell-derived xenograft treated with P7170 (see Figure 3A). (PPTX 404 KB) [file 12943_2014_1461_MOESM4_ESM.pptx]

## Slide 1
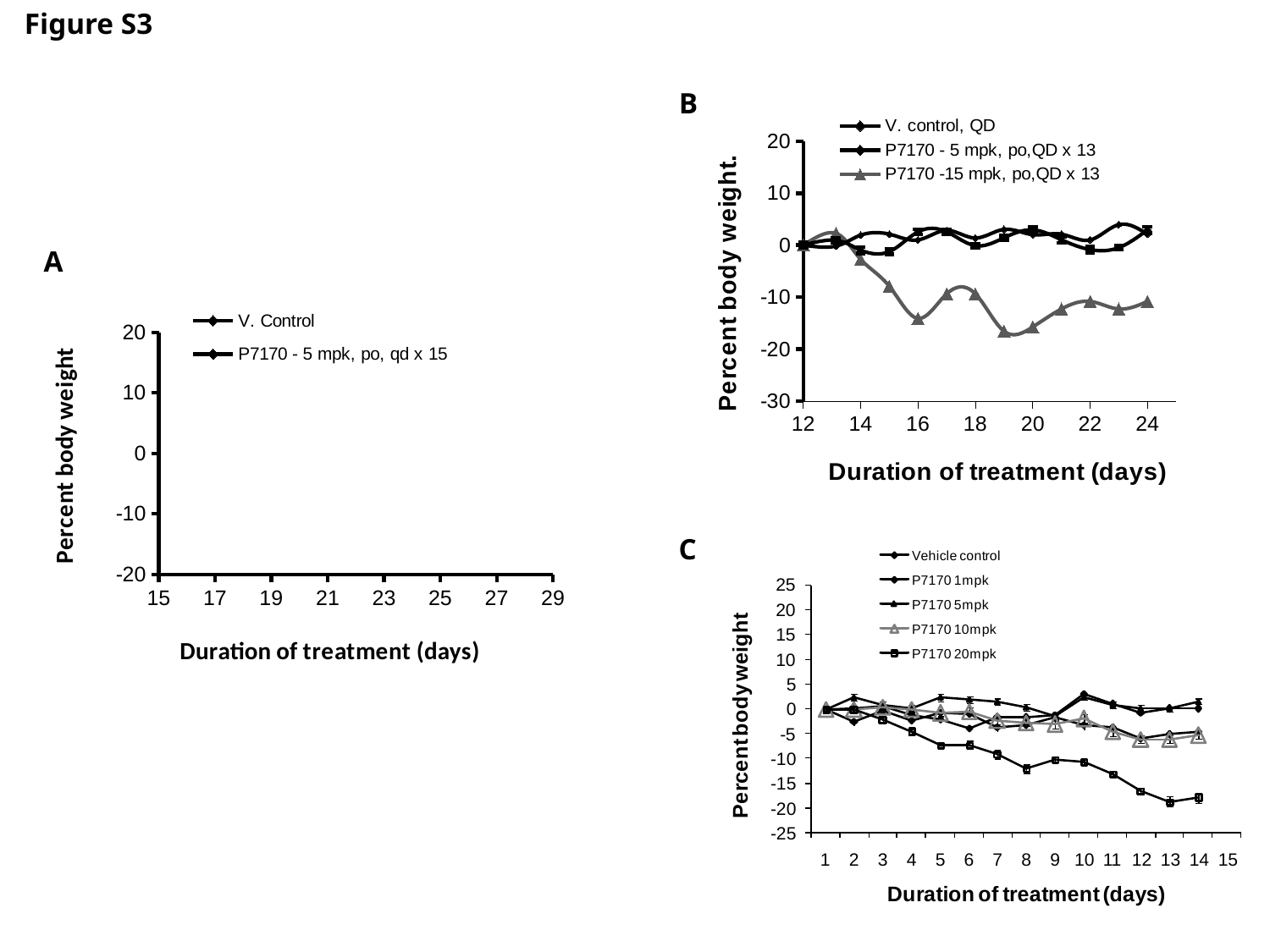

Figure S3
B
### Chart
| Category | V. control, QD | P7170 - 5 mpk, po,QD x 13 | P7170 -15 mpk, po,QD x 13 |
|---|---|---|---|A
### Chart
| Category | V. Control | P7170 - 5 mpk, po, qd x 15 |
|---|---|---|C
