# Supplementary material for: P7170, a novel inhibitor of mTORC1/mTORC2 and Activin receptor-like Kinase 1 (ALK1) inhibits the growth of non small cell lung cancer
Source: Mol Cancer. 2014 Dec 2;13:259. doi: 10.1186/1476-4598-13-259 (PMC4289333; doi:10.1186/1476-4598-13-259)
Supplement: Supplementary file 5 — Additional file 5: Table S1: Summary of PK/PD study. Correlation analysis of tumor volume to P7170 concentration. E0 represents the level of biomarker in plasma and tumor at baseline i.e. when the concentration of drug in plasma and tumor is 0 (zero). IC50 represents the concentration of drug in plasma and tumor required to produce 50% of the maximal inhibition. (PPTX 64 KB) [file 12943_2014_1461_MOESM5_ESM.pptx]

## Slide 1
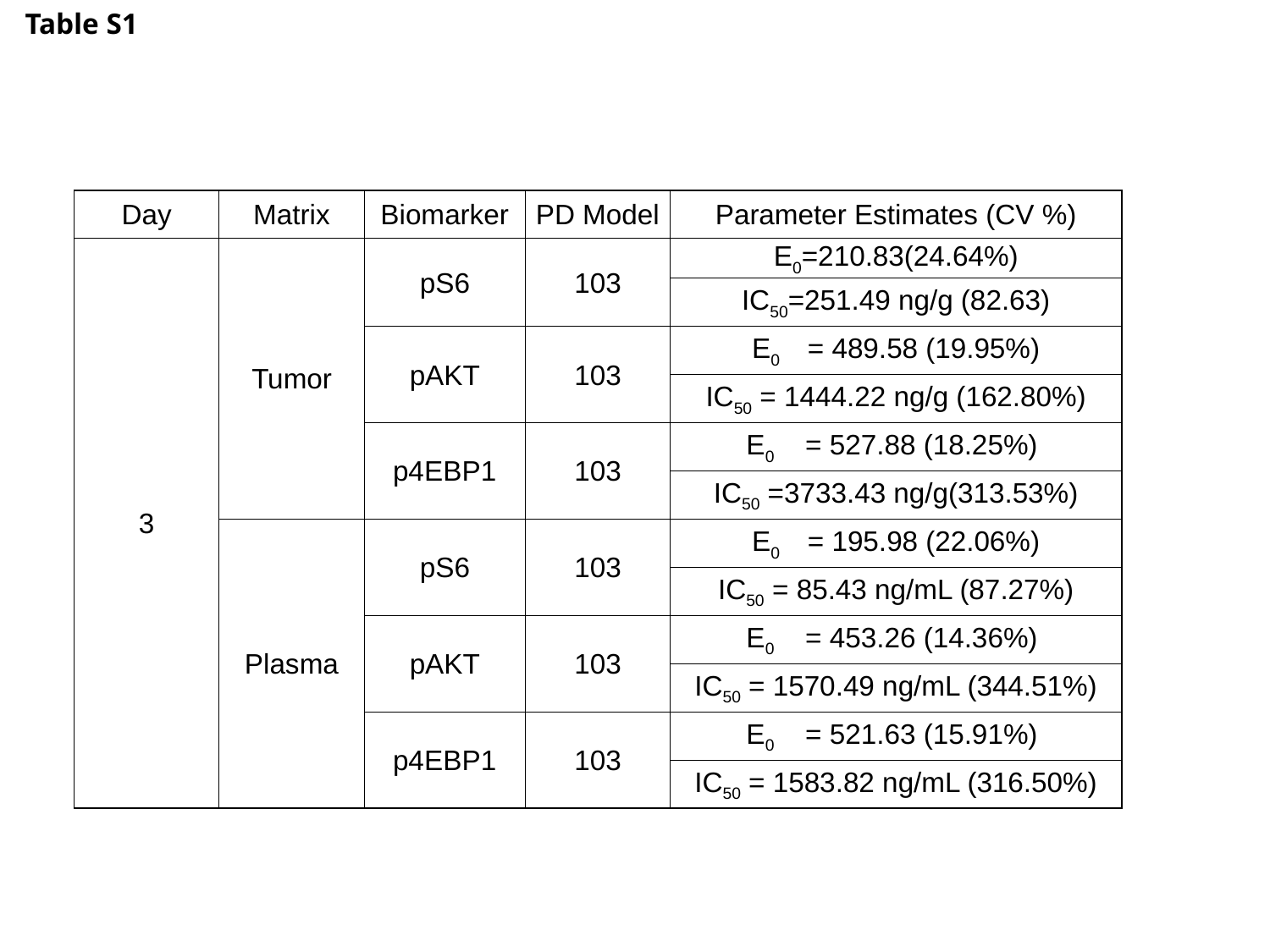

Table S1
| Day | Matrix | Biomarker | PD Model | Parameter Estimates (CV %) |
| --- | --- | --- | --- | --- |
| 3 | Tumor | pS6 | 103 | E0=210.83(24.64%) |
| | | | | IC50=251.49 ng/g (82.63) |
| | | pAKT | 103 | E0 = 489.58 (19.95%) |
| | | | | IC50 = 1444.22 ng/g (162.80%) |
| | | p4EBP1 | 103 | E0 = 527.88 (18.25%) |
| | | | | IC50 =3733.43 ng/g(313.53%) |
| | Plasma | pS6 | 103 | E0 = 195.98 (22.06%) |
| | | | | IC50 = 85.43 ng/mL (87.27%) |
| | | pAKT | 103 | E0 = 453.26 (14.36%) |
| | | | | IC50 = 1570.49 ng/mL (344.51%) |
| | | p4EBP1 | 103 | E0 = 521.63 (15.91%) |
| | | | | IC50 = 1583.82 ng/mL (316.50%) |
